# Predicting Achievable Fundamental Frequency Ranges in Vocalization Across Species

**DOI:** 10.1371/journal.pcbi.1004907

**Published:** 2016-06-16

**Authors:** Ingo Titze, Tobias Riede, Ted Mau

**Affiliations:** 1 National Center for Voice and Speech, University of Utah, Salt Lake City, Utah, United States of America; 2 Department of Physiology, Midwestern University, Glendale, Arizona, United States of America; 3 Department of Otolaryngology-Head and Neck Surgery, University of Texas Southwestern Medical Center, Dallas, Texas, United States of America; University of California at Berkeley, UNITED STATES

## Abstract

Vocal folds are used as sound sources in various species, but it is unknown how vocal fold morphologies are optimized for different acoustic objectives. Here we identify two main variables affecting *range* of vocal fold vibration frequency, namely vocal fold elongation and tissue fiber stress. A simple vibrating string model is used to predict fundamental frequency ranges across species of different vocal fold sizes. While *average* fundamental frequency is predominantly determined by vocal fold length (larynx size), *range* of fundamental frequency is facilitated by (1) laryngeal muscles that control elongation and by (2) nonlinearity in tissue fiber tension. One adaptation that would increase fundamental frequency range is greater freedom in joint rotation or gliding of two cartilages (thyroid and cricoid), so that vocal fold length change is maximized. Alternatively, tissue layers can develop to bear a disproportionate fiber tension (i.e., a ligament with high density collagen fibers), increasing the fundamental frequency range and thereby vocal versatility. The range of fundamental frequency across species is thus not simply one-dimensional, but can be conceptualized as the dependent variable in a multi-dimensional morphospace. In humans, this could allow for variations that could be clinically important for voice therapy and vocal fold repair. Alternative solutions could also have importance in vocal training for singing and other highly-skilled vocalizations.

## Author Summary

Mammals, birds, and reptiles vocalize (make sounds with vocal cords). Various species, and individuals within the species, are identified by pitch, loudness, and other acoustic features they can build into a repertoire of rhythmic and melodic patterns. Range of pitch, or more precisely fundamental frequency, is required to produce a variety of patterns. Whereas the average fundamental frequency is predictable by body size, the range of fundamental frequency depends on two different factors. The first is a freedom of movement factor–how much vocal cord length change can be produced by muscles that rotate or glide cartilages in the larynx. The second is a molecular composition factor–how much collagen density can be produced in the vocal cord ligament. Development and evolution has not been uniform with regard to these factors, suggesting that alternative choices are available for growth, training, and repair.

## Introduction

A biological trait is usually the result of a trade-off between different selective forces and constraints [1]. Vocal behavior is no exception, and one important set of constraints is related to the mechanism of sound production. In order to understand the design of vocal organs (larynx and syrinx in vertebrates), investigators have often focused on size as the primary determining factor of fundamental frequency and acoustic power produced by a sound source. In fact, a number of size-dependent factors are responsible for the observation that species of larger body sizes tend to produce lower frequencies[2],[3], yet some observations cannot be explained by vocal fold size alone. First, the relation between fundamental frequency (*f*_o_) and body size appears uncoupled within some species [4],[5],[6]. Considering that vocal fold size remains closely linked to body size, other mechanisms must facilitate the *f*_o_ variations. Second, vocal fold morphology in the mammalian larynx [7],[8] and labial morphology in the avian syrinx [9] vary greatly within and among species. Mechanical properties, a direct consequence of morphological design, also show a large variation and contribute to vocal differences within and between species [10],[11],[12],[8]. Third, the exceptionally large *f*_o_ range that some species rely on to generate large vocal versatility cannot be explained by size [11]. Here we present predictions from vibrating string theory that offer an explanation for why a larger than expected range of *f*_o_ can be achievable in large and small species.

If all species had the same tissue construct and the same ability to strain the vocal folds, then a vibrating string model would predict a larger *f*_o_ range (in Hz) for smaller animals, as will be shown. However, if the range is expressed in high/low ratios, or octaves, the range is normalized across species. It will be shown that, additionally, there is a large variation in this high/low ratio prediction because material properties are not the same and the ability to strain vocal fold tissues is also not the same.

The mechanism for achievement of a large *f*_o_ range in animals stands in stark contrast to the design of man-made musical string instruments, which utilize multiple strings to cover a wide pitch range. Violins have four strings, classical guitars have six, and pianos have eighty-eight, which are either single, doubled, or tripled. With these multiple strings, violins and guitars can produce on the order of 4–5 octaves of pitch range and a piano can produce a little over 7 octaves. Vocalizations in mammals [13],[14],[15],[16],[17],[18],[19] are generated by airflow-induced vibrations of vocal folds or labia, respectively. Humans, other mammals, and birds can produce 3 octaves, and in some cases 4–5 octaves, with a single pair of vocal folds in the larynx or labia in the syrinx. Vocal folds are basically the equivalent of one double string. What are the properties of these folds or labia that produce such versatile biological “strings”? We show here that geometry plays a role, but the dominant factor is the molecular structure of laminated tissue that can generate orders of magnitude variation in fiber tension.

The morphology of vibrating vocal fold tissue in the larynx is sufficiently complex that voice scientists and clinicians have debated for decades whether “vocal fold” or “vocal cord” is the best descriptor. Prior to Hirano’s [7] pioneering work, the term vocal cord was most prevalent, but it was understood that only the vocal ligament, a portion of the entire tissue construct, was cord-like. In human speech, the ligament is not under much tension, making the entire system fold-like in the sense that the superior portion folds over the inferior portion in vibration. Simple mechanical models have been of the *mass-spring* type to represent folding tissue,[20] but a *vibrating string* model was also introduced [21], [22].

**Fig 1:**
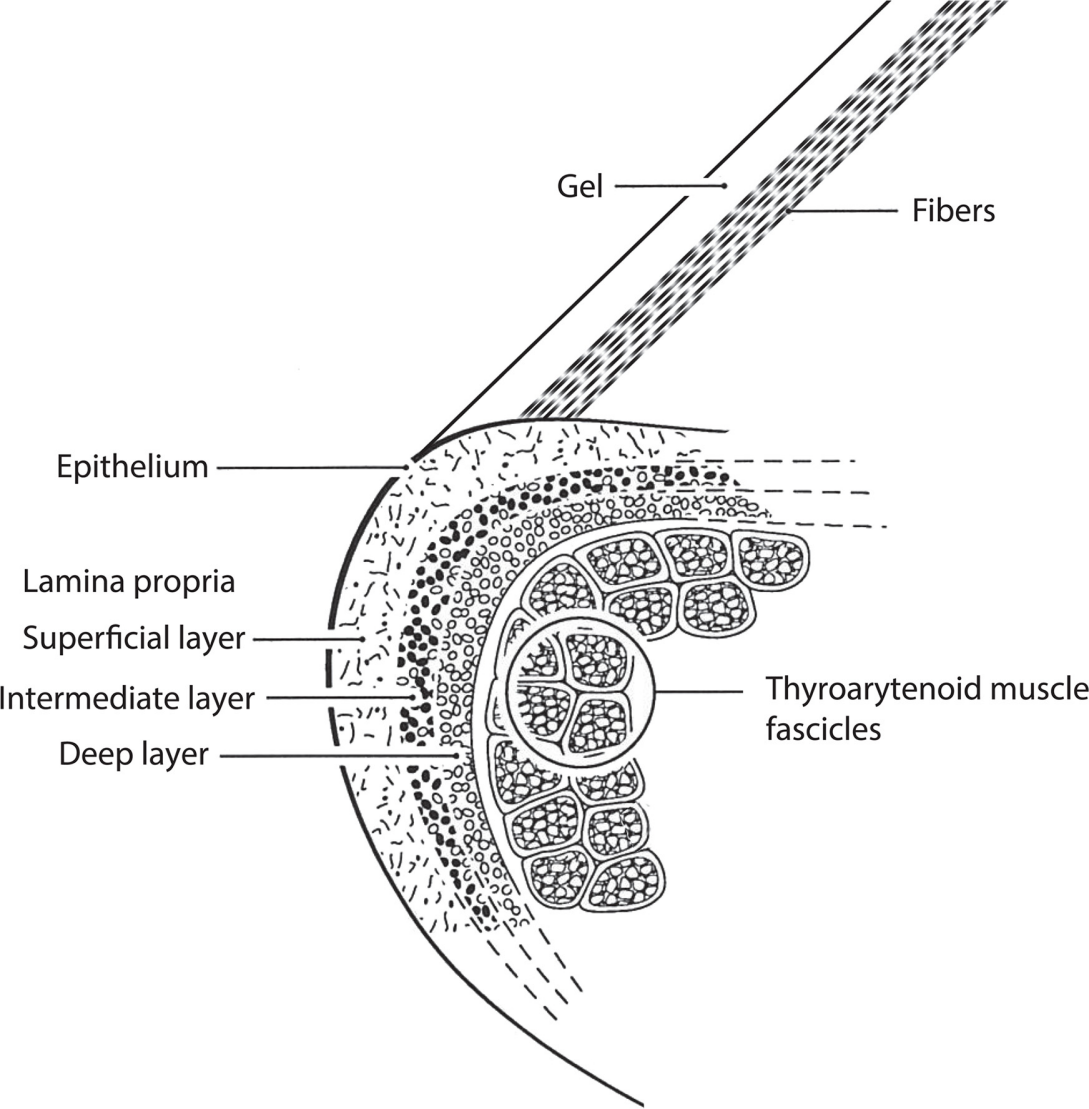
Schematic representation of vocal fold tissues, indicating three main layers: epithelium, lamina propria, and muscle. The lamina propria is further differentiated into a superficial, an intermediate and a deep layer. The intermediate and deep layers constitute the vocal ligament.

The conceptualization of a *fiber-gel* construct, not claimed here to be novel, embraces both the fold and the string construct (Fig 1). The ground substance is a viscoelastic continuum in the form of a homogenous, isotropic gel, similar to the vitreous humor in the eye. With the inclusion of directional fibers in multiple layers (collagen and elastin in the lamina propria and muscle fibers in the thyroarytenoid muscle), the construct develops into an adult human vocal fold. The development is gradual, however, and is likely influenced by vocal demand. At birth, the vocal fold consists of a single layer of ground substance (gel) with sparse fibers randomly oriented [23]. Through childhood and puberty, the gel develops into multiple morphological layers of tissue [26]. The superficial layer of the vocal fold lamina propria remains mostly ground substance (gel-like), whereas the intermediate and deep layers develop into elastin and collagen fibers aligned in a ventral-dorsal direction [24], [25], [26]. The fibers originate and insert on cartilages in the larynx (not shown) that can be moved by laryngeal muscles. The *moving boundaries* apply variable tension to the fibers.

## Methods

The main difference between multiple parallel strings on a violin and multiple “strings” embedded in vocal fold ground substance is the amount of mechanical coupling between the strings. The fibers cannot vibrate independently. There are cross-links in the form of an elastic matrix and there are proteoglycans and glycoproteins that fill the spaces in the form of a viscous liquid, leaving no air spaces between any of them. Such a viscoelastic medium, i.e., a laminated fiber-gel system, is subject to the laws of continuum mechanics. However, when the fibers of one layer are under considerable tension, the layer can be considered a “thick string vibrating in a viscous soup.” The string modes of vibration then dominate over the gel modes of vibration [27]. Here we consider such a simplified string model to be appropriate because range of fundamental frequency is largely determined by the fiber component. Small variations near the lower bound of *f*_o_ are determined by the combined viscoelastic properties of the gel and the fibers, but these variations contribute to a small part of the total range of normal mode frequencies [27].

### How is vibration frequency controlled with tissue fibers?

In a string fixed at both ends and under tension, the fundamental frequency of the dominant mode of vibration is

$$f_{o}=\frac{1}{2L}\sqrt{\frac{\mu^{\prime }}{\rho }}\;,$$ (1)

where *L* is the length of the string, *μ′* is the combined shear and tensile stress for vibrational displacement transverse to the string, and *ρ* is the tissue density. Density is a constant in soft tissue (about 1.04 g/cm^3^), which leaves control of *f*_o_ for any fibrous layer to *L* and *μ′*. In man-made string instruments, length is either held constant (e.g., piano) or varied with finger position (violin or guitar). In vocal folds, length can only be varied by moving boundary cartilages, which means that individual layers cannot be lengthened or shortened independently. Thus, with one common elongation, fiber stress *μ′* becomes the critical variable for *f*_o_ control between layers.

Based on Eq (1), the total variation in *f*_o_ can be written as

$$\Delta f_{o}=\frac{\partial f_{o}}{\partial L}\;\Delta L\;+\frac{\partial f_{o}}{\partial \mu^{\prime }}\;\Delta \mu^{\prime }$$ (2)

which after partial differentiation yields the expression

$$\Delta f_{o}=f_{o}\left[-\frac{\Delta L}{L}+\frac{1}{2}\frac{\Delta \mu^{\prime }}{\mu^{\prime }}\right]$$ (3)

Here we see that an absolute frequency range Δ*f*_o_ (in Hz) varies directly with *f*_o_. If the terms in brackets were equal across species, smaller species with higher mean *f*_o_ would have larger changes in *f*_o_. The above expression also shows that a positive change in fiber stress Δ*μ′*/*μ′* must overcome the negative change in strain Δ*L*/*L* if a positive change Δ *f*_o_ is to occur.

Non-muscular tissue layers, known as the lamina propria in the vocal folds, can experience an increase in *μ′* only with an increase in length. The length-tension curve must be highly nonlinear for a large *f*_o_ range. The degree of nonlinearity is related directly to the desired *f*_o_ range. Stress-strain curves of the vocal ligament are typically exponential [28],[10],[12], of the form

μ′=AeB(L−Lo)/Lo, (4)

where *A* and *B* are empirically-determined constants, *L* is an arbitrary length, and *L*_o_ is a reference length. According to Eq 1, two fundamental frequencies are related as

$$\frac{f_{o2}}{f_{o1}}={\left(\frac{L_{2}}{L_{1}}\right)}^{-1}e^{\frac{1}{2}B\left(L_{2}-L_{1}\right)/L_{o}}\;.$$ (5)

**Fig 2:**
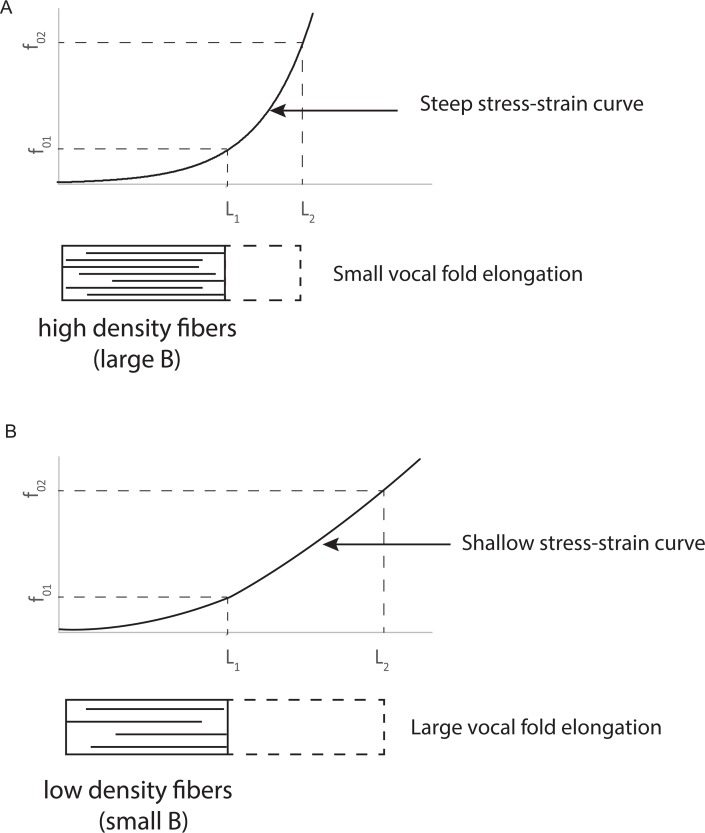
Two contrasting cases for obtaining a similar *f*_o_ range. (a) A steep stress-strain curve with small elongation, and (b) a shallow stress-strain curve with large elongation.

Note that for *B* = 0 (constant fiber stress at all lengths), the fundamental frequency ratio is inversely related to vocal fold length ratio. This is the general size principle. The larger the animal, the longer the vocal folds and the lower the frequency if stress is kept constant. The reference length *L*_*o*_ is generally taken as the *in situ* cadaveric length for measurement purposes. From this reference length, the length for phonation can be increased and decreased on the order of ± 50%, but typically more like ± 30%, as will be shown later. Fig 2 shows two contrasting cases of how the same *f*_o_ range can be produced. In Fig 2(A) the stress-strain curve is steep, with a large *B* value, and the elongation is small. In Fig 2(B) the stress-strain curve is shallow, with a small *B* value, but the elongation is large. Anatomically and physiologically, the trade-off is between range of motion between cartilages versus fiber tension in the vocal folds.

**Fig 3:**
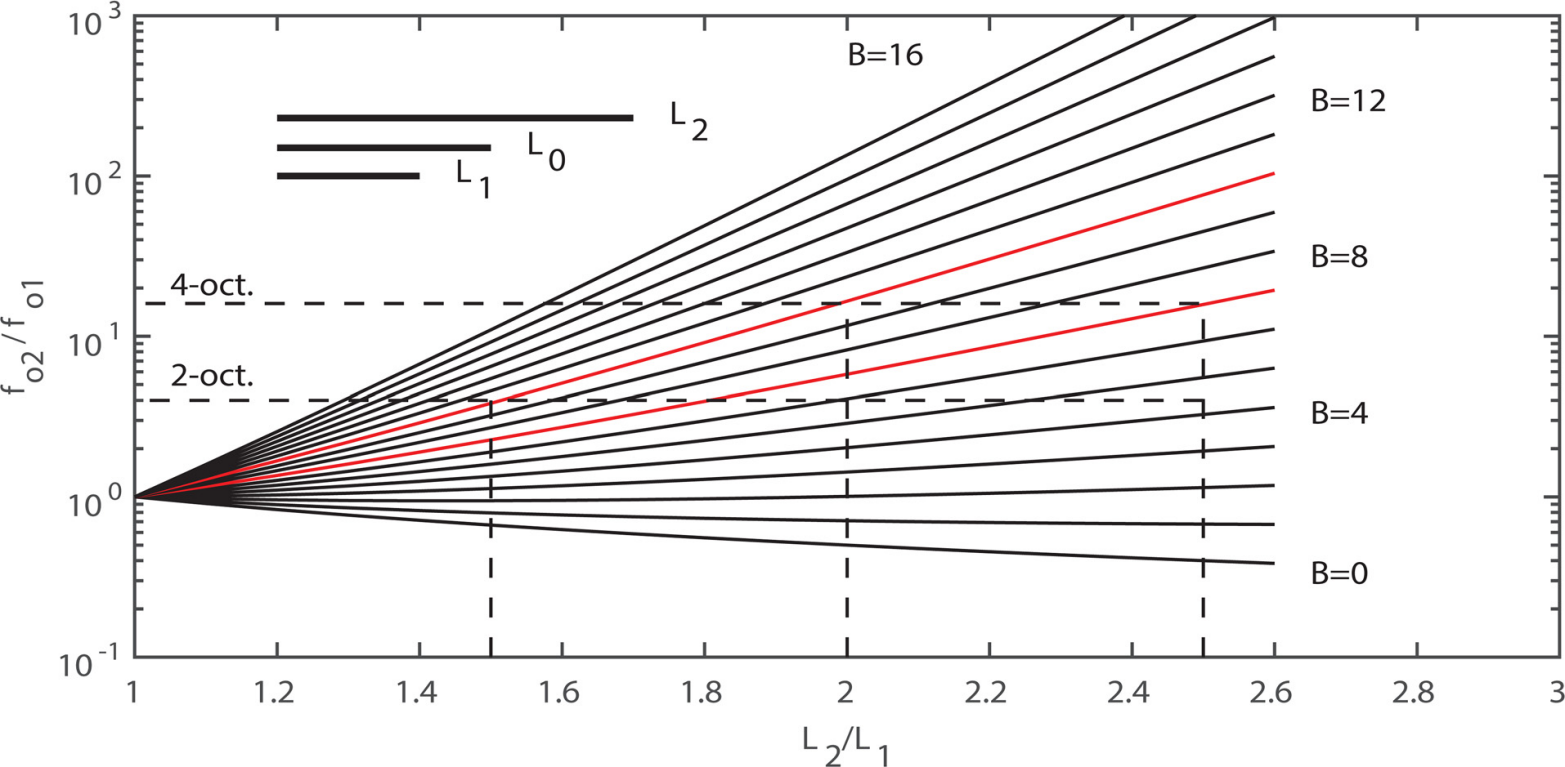
Relations between logarithmic fundamental frequency ratio (high/low) and the respective vocal fold length ratio (long/short) for vocal fold tissue characterized by different B-values (from Eq 3). A value of *L*_1_ = 0.7*L*_0_ was assumed.

Fig 3 shows a plot of Eq 5, with an assumption that *L*_1_ = 0.7*L*_*o*_. The *L*_2_/*L*_1_ ratio is plotted on the horizontal axis and the *B* value is the parameter. We will show that a value of 2 for *L*_2_/*L*_1_ appears to be a typical limit in humans (a length change from *L*_*1*_ = 0.7*L*_o_ to *L*_*2*_ = 1.4*L*_o_ in the phonation range). The value of *B* is determined by the density of collagen fibers that can be packed into the ligament layer of tissue. According to Fig 3, a four octave range (*f*_o2_/*f*_o1_ = 16) requires an exponent value of *B* = 10 if the *L*_2_/*L*_1_ ratio is 2, as the intersection of the middle vertical dotted line and the upper red solid line shows in the diagram. If *L*_2_/*L*_1_ is restricted to 1.5, only a 2 octave range is obtained with *B* = 10 (left-most dotted line). However, a 4 octave range would be achievable with *B* = 7 (lower red line) if *L*_2_/*L*_1_ were 2.5 (right-most vertical dotted line). Thus, frequency range hinges on two variables, ability to change vocal fold length and nonlinearity of the dominant fiber stress-strain curve. Some data will now be given from various species.

### Measurements from human larynges

Vocal fold length change with *f*_o_ has been quantified in several investigations. [29] used stereo videoscopy to measure the membranous vocal fold length during phonation in 4 female and 3 male human subjects. For the males, *L*_1_ averaged 0.77 cm and *L*_2_ averaged 1.3 cm, such that *L*_2_/*L*_1_ was 1.7. For the females, *L*_1_ averaged 0.71 cm and *L*_2_ averaged 1.1 cm, such that *L*_2_/*L*_1_ was 1.5. The fundamental frequency ranged on the order of 100–500 Hz for the males and 130–800 Hz for the females. Thus, a 2 ½ octave *f*_o_ range was achieved with the *L*_2_/*L*_1_ ratios of 1.7 for males and the *L*_2_/*L*_1_ ratio of 1.5 for females. Fig 1 would predict values of *B* in the 8–9 range if the string model applies to the combination of fibrous tissue layers (ligament and muscle). Later reports of measurements of *B* will confirm this range of values. In so-called “falsetto” register, the vocal ligament (intermediate and deep layers of the lamina propria) dominates in *f*_o_ control [14]. In this falsetto region, Nishizawa et al. measured an approximate 1-octave *f*_o_ range with an *L*_2_/*L*_1_ ratio of no more than 1.2. According to Fig 3, this would require a value of *B* of about 10–12. Min et al. [28] measured a value of 9.7 in human ligaments (8 specimens from three males and two females, left and right averaged). Chan et al. [10] measured values of 9.4 for males and 7.6 for females.

A more recent study by Cho et al. [30] on vocal fold length change in humans used an ultrasonic imaging technique to follow anterior and posterior landmarks on the vocal folds. Results showed that *L*_1_ = 1.47 cm and *L*_2_ = 2.0 cm for males for low and high pitch, with *L*_2_/*L*_1_ = 1.4. For females, *L*_1_ = 1.14 and *L*_2_ = 1.65 also yielded an *L*_2_/*L*_1_ ratio of 1.4. This is a little less than the ratios of 1.7 for males and 1.5 for females reported by [29]. The small discrepancy is probably related to a smaller *f*_o_ range in the Cho et al. study, but unfortunately the *f*_o_ ranges were not reported.

### Measurements from non-human species

Measurements for length change versus fundamental frequency are also available from studies using excised larynges [30], [31], [32]. For example, excised domestic dog (*Canis familiaris*) larynges were vibrated on a laboratory bench with an artificial air supply [31]. Self-sustained vocal fold oscillation was achievable from *L*_1_ = 0.5 cm to *L*_2_ = 1.2 cm, but these lengths were produced mechanically rather than by muscle control. The corresponding *f*_o_ range was 50 to 230 Hz, somewhat greater than 2 octaves. For this large *L*_2_/*L*_1_ ratio of 2.4, the value of *B* from Fig 3 would be predicted to be about 6.0. Some dogs do not have a vocal ligament, but measurements on canine mucosa produced a value of *B* = 4.4 and measurements on canine thyroarytenoid muscle fibers yielded a value of *B* = 6.5 [27]. Given that the thyroarytenoid muscle is the fibrous layer in vibration, it would dominate the *f*_o_ range. The predicted and measured value of *B* are therefore in agreement.

Table 1 shows measured stress-strain relations for various mammalian species, [8], [10–12], [33–42]. In some cases, the frequency ranges are shown. Note that the rhesus monkey has an approximate four-octave range (100–1800 Hz). With values of *B* = 16.2 for males and 12.9 for females, this range is achievable with a modest *L*_2_/*L*_1_ ratio of about 1.6 for males and 1.7 for females according to Fig 3.

**Table 1:** Raw data of body mass, vocal fold length (*L*_0_), stress-strain relationship for vocal fold tissue, and average fundamental frequency range. Vocal fold lengths were measured in specimen available to us (unpublished data), except for the African elephant. The variable *ε* = (*L*-*L*_0_) / *L*_0_.

Species	Body mass (kg)	L_0_ (mm)	Exponential model of stress strain relation (Eq 2)	*f*_o_ range of natural vocal repertoire (Hz)	Sources
Greater horseshoe bat	0.02	1		2000–80000	[33]
House Mouse	0.05	1		Audible sounds 1000–9000	Pers. observation
Laboratory Rat	0.4	2	male: 0.5*e*^4.4*ε*^	audible sounds produced by males and females: 500 to 6000	[34]
Guinea pig	0.8	2		250–4000	[35]
Human	male 75, female 60	male 16, female 10	male: 4.0*e*^9.4*ε*^, female: 5.5*e*^7.6*ε*^	male: 90–450, female: 120–800	[36], [10], [28]
Rhesus monkey	male 6.4, female 5.1	male 8.3, fem. 7.8	male: 1.1*e*^16.2*ε*^, female: 2.5*e*^12.9*ε*^	males and females: 100–1800	[37], [38]
Raccoon	5	4		200–4000	[39]
Domestic Dog	10	8		60–1500	[40]
Rocky Mountain elk	male 250, Female 150	Male 31, Fem. 29	male: 3.3*e*^6.5*ε*^, female: 3.1*e*^5.9*ε*^	males and females: 100 to 2400	[11]
Mule deer	male 80, female 65	male 24, female 21	male: 3.8*e*^8.2*ε*^, female: 2.9*e*^7.9*ε*^		[12]
Grevy’s Zebra	200	29			[41]
Domestic cow	400	35			[15]
African Lion	250	38	male: 0.7*e*^8*ε*^	males and females: 20 to 250	[8]
Siberian Tiger	300	40	male: 0.8*e*^8.5*ε*^	males and females: 20 to 250	[8]
Giraffe	1000	40			pers. observation
African Elephant	5000	100			[42]

**Fig 4:**
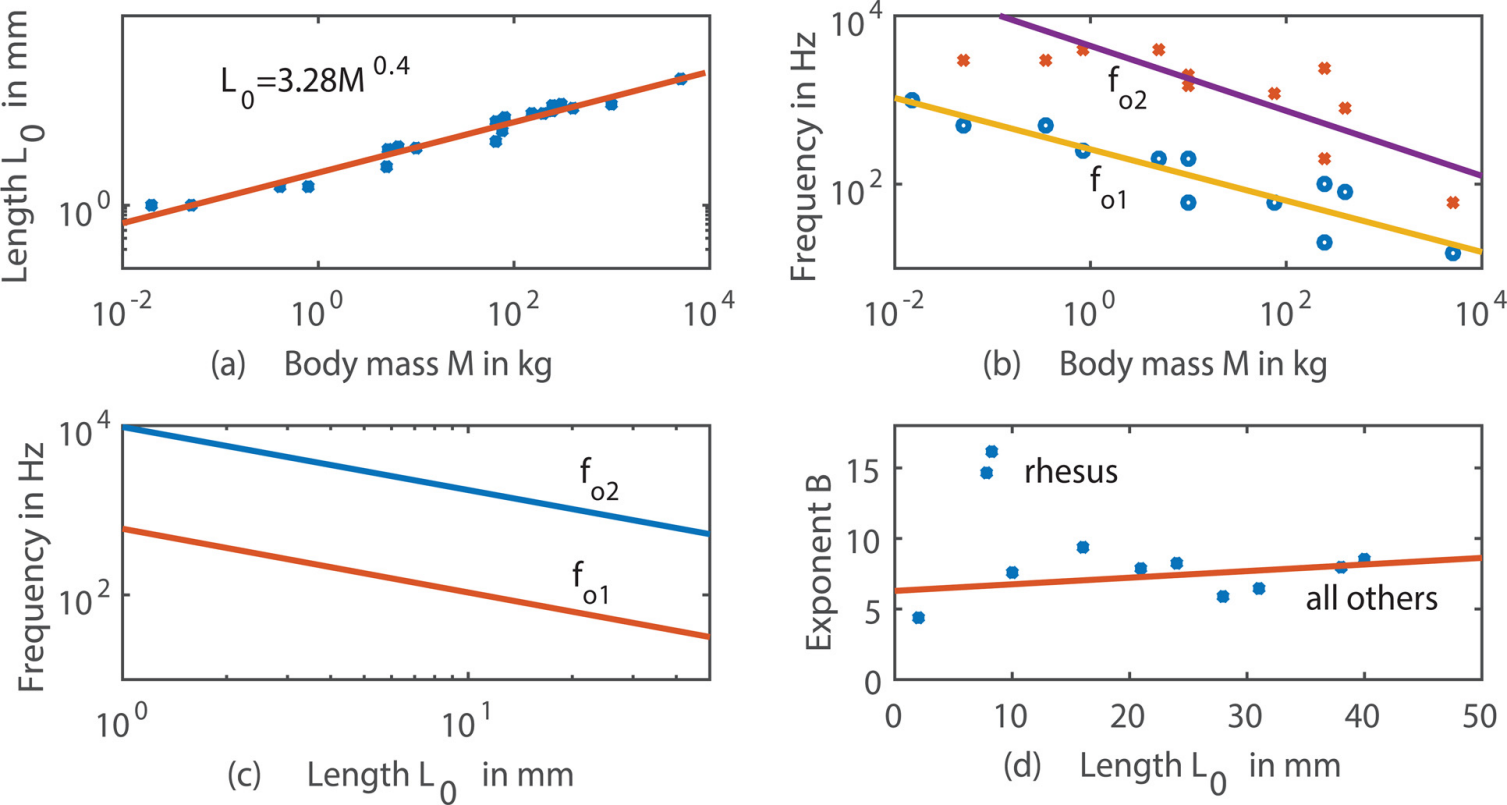
**Empirical data from Table 1, (a) cadaveric vocal fold length *L*_0_ versus body mass, (b) minimum and maximum fundamental frequency versus body mass, (c) derived minimum and maximum fundamental frequency versus cadaveric vocal fold length *L*_0_, (d) *B*-value versus cadaveric vocal fold length *L*_0_; the trend line was calculated without one outlier (rhesus monkey)**.

Fig 4(A) shows measurements of vocal fold cadaveric length *L*_*o*_ as a function of body mass *M* for fourteen species in Table 1 (mouse to giraffe, in some cases both male and female). Note the general increase in *L*_*o*_ with size, plotted logarithmically with the regression line

$$L_{0}=3.28\;M^{0.4}\;r^{2}=0.96\;\text{p}<0.001$$ (6)

The regression is a very tight fit over a length range of 1–40 mm and a body mass range of .05–1000 kg, reinforcing the earlier claim that vocal fold length and body mass are tightly related.

Fig 4(B) shows high and low values of *f*_o_ as a function of body mass (size). There is a general decrease of *f*_o1_ and *f*_o2_ with mass, expressed by the following regression lines

$$f_{\text{o}2}=63196M^{-0.386}\;\text{Hz}\;r^{2}=0.70\;\text{p}<0.01$$ (7)

$$f_{\text{o}1}=2135.7M^{-0.305}\;\text{Hz}\;r^{2}=0.85\;\text{p}<0.001$$ (8)

It is clear, however, that much greater variability is associated with these frequency trends, suggesting that factors other than body mass play a role in fundamental frequency prediction. Combining Fig 4(A) and 4(B) by eliminating the mass variable from the regression equations, an inverse relation between *f*_o1_ and *f*_o2_ with cadaveric length *L*_*o*_ is obtained, as shown in Fig 4(C). This inverse relation is in agreement with the *B* = 0 curve in Fig 3, showing only the length dependence on *f*_o_. Note that the *range* of *f*_o_, if expressed logarithmically as a ratio *f*_o2_/*f*_o1_ rather than a difference *f*_o2_ –*f*_o1,_ is essentially a constant. This is a strong validation of the simple vibrating string model (Eq 5). Taking the ratio of Eq (7) to Eq (8) yields the number 12.0, which constitutes about 3.5 octaves as an average across species.

Empirical data for exponent *B* versus *L*_*o*_ are shown in Fig 4(D). Omitting the one outlier (the rhesus monkey), a mild trend is quantified by the relation

$$B=6.285\;+\;0.0468\;L_{\text{o}}\;r^{2}=0.15\;\text{p}<0.3\;\text{rhesus monkey excluded}$$ (9)

However, when the outlier is included, the trend disappears. Thus, with the sparsity of data available across species, it is not possible to assert whether or not there is an increase in *B* with longer vocal folds. What is important to note, however, is the large variation in *B* across species. Since *B* is an exponent, a range of 3–15 leads to orders-of-magnitude variations in frequency range.

**Fig 5:**
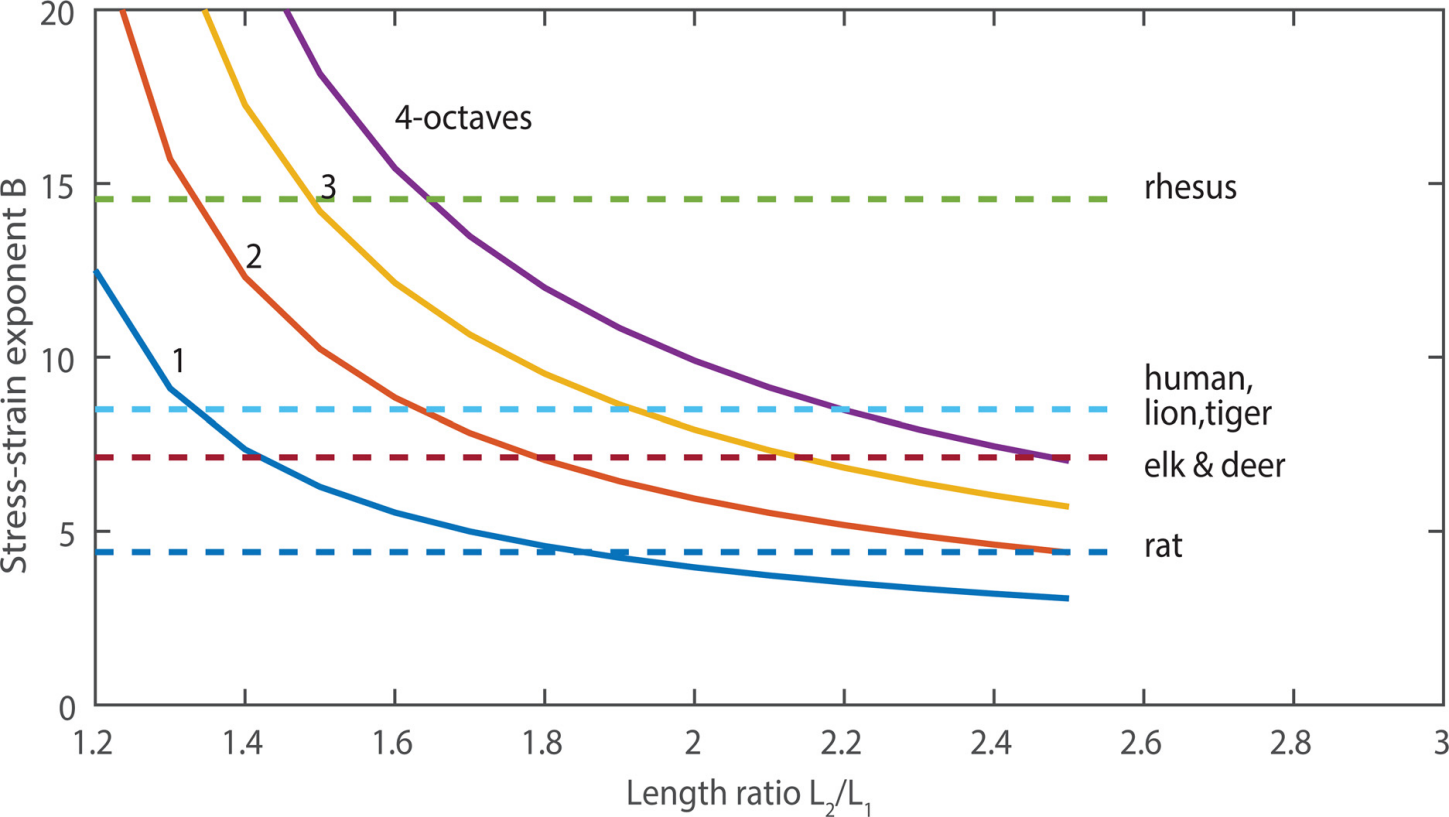
Contour plot of predicted fundamental frequency range (high/low, *f*_o2_/*f*_o1_ ratio) for morphological variables *B* and *L*_2_/*L*_1_. The range depends on two important factors: the rotational flexibility of the laryngeal framework, which facilitates *L*_2_/*L*_1_; and the *B* value that quantifies the tissue stress response to elongation. For a given B value, a larger fundamental frequency range can be achieved with greater rotational flexibility. For a given *L*_2_/*L*_1_ ratio, a larger frequency range can be achieved with a greater *B* value. Note that the changes in the *B* value are not large to achieve a larger frequency range for a given a given *L*_2_/*L*_1_ ratio.

With this empirical relation between *B* and *L*_*o*_, a better *f*_*o*_ range prediction can be made with Eq 5. If we continue to assume that *L*_1_ = 0.7 *L*_*o*_, as in humans, then Fig 5 shows a contour plot of the *f*_o2_/*f*_o1_ range achievable in octaves. The two morphological variables are *B* on the vertical axis and *L*_2_/*L*_1_ on the horizontal axis. The figure shows that a greater *f*_o_ range is attainable with either greater *B* or greater *L*_2_/*L*_1_. The empirical *B* values allow some species to be identified on the figure. The greater the *B* value, the smaller *L*_2_/*L*_1_ needs to be to achieve a large *f*_o_ range. Conversely, the larger *L*_2_/*L*_1_ is, the smaller *B* needs to be to achieve a large *f*_o_ range. For example, the male rhesus monkey requires only an *L*_2_/*L*_1_ ratio of 1.6 for a 4-octave range. Humans, lions, and tigers require an *L*_2_/*L*_1_ ratio of about 2.2 for a 4-octave range. For animals that scream or roar, a larger *B* value may be a protective requirement for greater vibrational amplitude and vocal fold collision.

## Results and Discussion

A simple theory of the *range* of fundamental frequency *f*_o_ achievable in various species has been proposed. Laryngeal size, and specifically vocal fold length, is a good predictor of mean *f*_o_, but a poor predictor of *f*_o_ *range*. When vocal fold tissues become layered and tissue fibers assume a ventral-dorsal direction, the layer with the densest and stiffest fiber composition produces string-like vibration and determines the *f*_o_ range. This can be a vocal ligament or a layer of muscle fibers. The stress-strain curve of the fibrous layer must be highly nonlinear to overcome the natural tendency for *f*_o_ to decrease with increased length. For an exponential stress increase with a factor *e*^*Bɛ*^, where *ε* is the strain (fractional length change) and *B* is a stiffness constant, a range of values 5 < *B* < 15 can produce a 4–5 octave *f*_o_ range with greater or lesser length change. If the laryngeal framework mechanics allows a large length change, on the order of ± 50% from the resting length, *B* values on the order of 5–10 can produce the 4–5 octave range. If the larynx is restricted in its range of motion such that only a ± 20% length change is possible, a value of *B* on the order of 10–15 is necessary to obtain a 4–5 octave range. A laryngeal adaptation for greater length change is greater rotation or gliding between cartilages that anchor the ends of the vocal folds. Alternatively, a tissue layer that can bear a greater tension (i.e., a ligament with high density collagen fibers) can also increase the fundamental frequency range and thereby allow vocal versatility. As a consequence, fundamental frequency can become uncoupled from size. Two large frequency ranges produced by two species can overlap even if the two have dramatically different body sizes.

The proposed framework for fundamental frequency range regulation has three important implications. First, voice production is an example of “many-to-one” mapping, which occurs when the functional property of interest depends on more than one underlying morphologic parameter [43]. In the cases of voice fundamental frequency, the parameters include laryngeal framework mechanics and all variables affecting the *B* value, i.e. the number and depth of vocal fold tissue layers, vocal fold boundary geometry, and tissue fiber stress. Consequently there are surfaces in a morphospace that represent functionally neutral variations, which means that morphological diversity between vocal folds of different species is not necessarily indicative of functional diversity. The evolution of vibrating tissue design in laryngeal or syringeal sound sources may lead to different morphologies that function similarly. For example, multilayered characteristics have been described in vocal folds of different mammals[8],[12],[44] as well as in alligators [45] and even within the oscillating tissue masses (“*labia*”) in the avian vocal organ, the syrinx [9]. Laryngeal design across mammals is morphologically distinct in each species but fundamental frequency remains overlapping. Findings in excised mammalian larynges [32] or the excised avian syrinx [19] suggest that multiple activation patterns of intrinsic muscles of the larynx and syrinx, respectively, produce a redundant output, i.e. they can facilitate similar vocal frequencies. In a complex laryngeal or syringeal cartilaginous framework, different muscle activations generate different tension settings of the oscillating tissue, yet in combination with the appropriate driving pressure, the soft tissue can vibrate at identical rates [46].

Our findings have a second, more practical implication as it pertains to the treatment of human voice disorders. The observation that multiple vocal fold morphologies can serve the same function, i.e. produce the same fundamental frequency, can be informative for surgical treatment of impaired vocal folds. Surgery to remove vocal fold lesions often results in irreparable loss of normal vibratory mucosa [47]. Restoration of normal human vocal fold morphology may not be feasible in many cases because the deficits are large. Our proposal that fundamental frequency range can be regulated through two distinct mechanisms, and its broader implication that multiple vocal fold morphologies can achieve the same vocal output, suggest vocal function may be restored with alternative strategies. Examples of alternative morphologies already exist in laryngeal surgery in which non-laryngeal tissue is used to restore voice production [48],[49],[50]. However, the concept of alternative morphologies as viable solutions has not been considered systematically in vocal fold repair and deserves further exploration. Computer simulation of voice production can provide the means for intelligent exploration of the vocal fold morphospace to search for viable alternatives. Simulations based on finite-element and finite-difference approaches have been reported over the past two decades [51],[52],[53],[54].

A single simulation produces one set of acoustic output variables given a defined input vocal fold morphology at a fixed subglottal pressure. A meaningful comparison between two different vocal fold morphologies should entail a range of possible acoustic outputs, given a clinically relevant range of subglottal pressures as well as a range of physiologic variations in the vocal fold morphologies. Such a comparison would entail thousands of simulation runs to fully cover the range of inputs. One approach to reduce the computational cost and to increase the efficiency of morphospace exploration is to combine a finite element model (FEM) voice simulation with multiobjective optimization [55]. This approach has been applied to vocal fold surgery simulation, in which the functional viabilities of two alternative vocal fold morphologies were demonstrated *in silico* [56].

Finally, the current findings relate well to vocal development and vocal training. If the density of collagen fibers in the vocal ligament is increased by exercise (frequent stretching), a speaker or a singer can increase the fundamental frequency range even if the laryngeal framework cannot be altered much due to tight spaces between cartilages. On the other hand, laryngeal massage and framework exercise could widen the spaces, allowing greater *f*_o_ range with existing molecular constructs. It appears that the development of a theory for fundamental frequency range regulation based on comparative data *across species in nature* is paramount to understanding possible intervention strategies for improving human communication.
